# Factors associated with postpartum complications: A systematic review to inform early detection strategies

**DOI:** 10.12688/f1000research.166397.1

**Published:** 2025-07-03

**Authors:** Elisa Ulfiana, Tandiyo Rahayu, Yuni Wijayanti, Dina Nur Anggraini Ningrum, Amalia Augustina Fadlilah, Faizul Hasan

**Affiliations:** 1 Public Health Doctoral Programs, Faculty of Medicine, Universitas Negeri Semarang, Semarang, Central Java, Indonesia; 2 Midwifery Program, Politeknik Kesehatan Kementerian Kesehatan Semarang, Semarang, Indonesia; 3 Public Health Master Program, Faculty of Medicine, Universitas Negeri Semarang, Semarang, Central Java, Indonesia; 4 Faculty of Nursing, Chulalongkorn University, Bangkok, Bangkok, Thailand

**Keywords:** Postpartum Complications; Maternal and Child Health; Health Inequities; Early Detection and Intervention; Sustainable Health Systems.

## Abstract

**Background:**

Complications during the puerperium present abnormal conditions that can jeopardize the health and well-being of both mother and newborn. These complications are significant contributors to maternal morbidity and mortality worldwide. This systematic review aims to identify and synthesize evidence on the factors associated with postpartum disease complications.

**Methods:**

This systematic review was conducted in accordance with the Preferred Reporting Items for Systematic Reviews and Meta-Analyses (PRISMA) guidelines. The protocol was registered in PROSPERO (CRD420251013692). A comprehensive search was performed using electronic databases, including Scopus and PubMed, to identify relevant studies published between 2014 and 2024. Eligible studies included full-text, peer-reviewed articles that used quantitative or qualitative designs and addressed postpartum complications in accordance with the PEO framework. Descriptive analysis was used to synthesize findings.

**Results:**

A total of 907 records were identified, and 13 studies met the inclusion criteria. The findings highlight a range of factors significantly associated with postpartum complications, including postpartum hemorrhage, maternal age >35 years, depressive and anxiety disorders, preeclampsia, multiple pregnancies, type of vaginal delivery, infant-related health concerns, childhood trauma, low social support, low educational attainment of both mothers and husbands, perceived social isolation, and marital dissatisfaction. While early detection and intervention were not directly evaluated in these studies, the identification of these risk factors suggests that targeted screening and supportive strategies may be beneficial in mitigating postpartum complications.

**Conclusions:**

This review identifies multiple psychosocial, demographic, and clinical factors associated with postpartum complications. These findings underscore the importance of early detection and comprehensive postpartum care to mitigate risks and improve maternal health outcomes.

## Introduction

The maternal mortality rate (MMR) refers to the number of women who die from pregnancy-related causes—excluding accidental or incidental causes—during pregnancy, childbirth, or within 42 days postpartum, per 100,000 live births. ^1^ Over 99% of maternal deaths from pregnancy-related complications occur in low- and middle-income countries. ^2^

Between 2000 and 2017, the global MMR declined by 38%, from 451,000 to 295,000 deaths. ^2^ Despite this progress, maternal mortality remains a critical global health challenge. The Sustainable Development Goals (SDGs) aim to reduce the global MMR to below 70 per 100,000 live births by 2030, alongside targets for neonatal and under-five mortality. ^2^ In 2020, nearly 800 women died every two minutes due to maternal causes, with 95% of these deaths occurring in developing countries—indicating that the global SDG target remains unmet. Indonesia ranks third in MMR among Southeast Asian countries.

The postpartum period is particularly high-risk, accounting for approximately 57% of maternal deaths globally. ^3^ Among these, 50% occur within the first 24 hours after delivery, 20% within the first seven days, and 5% between two and six weeks postpartum. ^4^ This period is vulnerable to complications such as postpartum hemorrhage, hypertensive disorders of pregnancy (HDP), anemia, malaria, helminth infestation, HIV, and preeclampsia—all of which significantly increase the risk of maternal mortality. ^5^

The Maternal Morbidity Measurement (MMM) framework offers a structured approach to analyzing postpartum complications by considering distant, near, and intermediate determinants of maternal health outcomes. ^6^ Integrated postpartum nursing care has proven effective in reducing complications, and timely intervention by skilled healthcare professionals before, during, and after childbirth is essential to prevent maternal deaths. ^2^ The quality of postpartum care is typically assessed by the coverage of comprehensive follow-up visits, with a recommended target of more than 90%. ^6^

Early detection, including screening in collaboration with family members, plays a vital role in preventing postpartum complications. Delays in recognizing danger signs and seeking timely care at referral facilities contribute significantly to adverse outcomes. ^7^ Screening for risk factors during the postpartum period facilitates early intervention and reduces the likelihood of severe complications. ^8^

Although several studies have explored factors associated with postpartum complications, findings have not yet been comprehensively synthesized. This systematic review aims to identify and analyze the factors associated with postpartum disease complications to support early detection and intervention strategies.

## Methods

### Data sources and search strategy

This study employed a systematic literature review methodology in accordance with the Preferred Reporting Items for Systematic Reviews and Meta-Analyses (PRISMA) guidelines. ^9^ The review followed the 27-item PRISMA checklist and utilized the flow diagram to guide study selection. The review protocol was registered with PROSPERO (CRD420251013692) and received ethical approval from the Research Ethics Committee of Universitas Negeri Semarang (Approval No. 332/KEPK/FK/KLE/2024).

The literature search was conducted using two major electronic databases: Scopus and PubMed. Articles published between 2014 and 2024 were considered. The search strategies are listed in Table 1.

**Table 1.** Search strategy.

Database	Keywords
PubMed	(“postpartum period”[MeSH Terms] OR “postpartum period”[All Fields]) OR (“postpartum woman”[All Fields]) AND (“early detection”[MeSH Terms] OR “early detection”[All Fields]) AND (“puerperal disorders”[All Fields])
Scopus	(“postpartum period”) OR (“postpartum woman”) OR (“partum period”) AND (“postpartum woman”) AND (“early detection”) AND (“puerperal disorders”)

### Study selection

Article selection was based on the Population, Exposure, and Outcome (PEO) framework. The population included postpartum women (puerperal mothers). The issue addressed factors contributing to postpartum complications, while the outcome focused on early detection of such complications. Eligible studies were limited to peer-reviewed full-text journal articles, either quantitative or qualitative in design, published in reputable international journals between 2014 and 2024. Articles that met these criteria were included following a four-stage PRISMA process: identification, screening, eligibility, and inclusion. All selected articles were extracted and synthesized using descriptive analysis.

### Data extraction

Two reviewers independently extracted relevant information from each study, including the names of authors, year of publication, study location, sample size, study design, and characteristics related to postpartum conditions. Any disagreement between the reviewers was resolved through discussion with a third author.

### Data synthesis

The methodological quality and risk of bias of the included observational studies were assessed using the Newcastle–Ottawa Scale (NOS). ^10, 11^ The NOS evaluates three domains: selection of study groups (maximum 4 points), comparability of groups (maximum 2 points), and exposure or outcome assessment (maximum 3 points), with a total possible score of 9 points. Based on the total score, studies were categorized into three quality levels: very high risk of bias (score 0–3), high risk of bias (score 4–6), and low risk of bias (score 7–9). Studies that scored below 5 points were considered low quality and excluded from the review. For each included article, extracted data included the authors, research objectives, study setting, sample size, study design, statistical analysis, and key findings. ^12, 13^

## Results

### Study selection and characteristics

**Figure 1.**
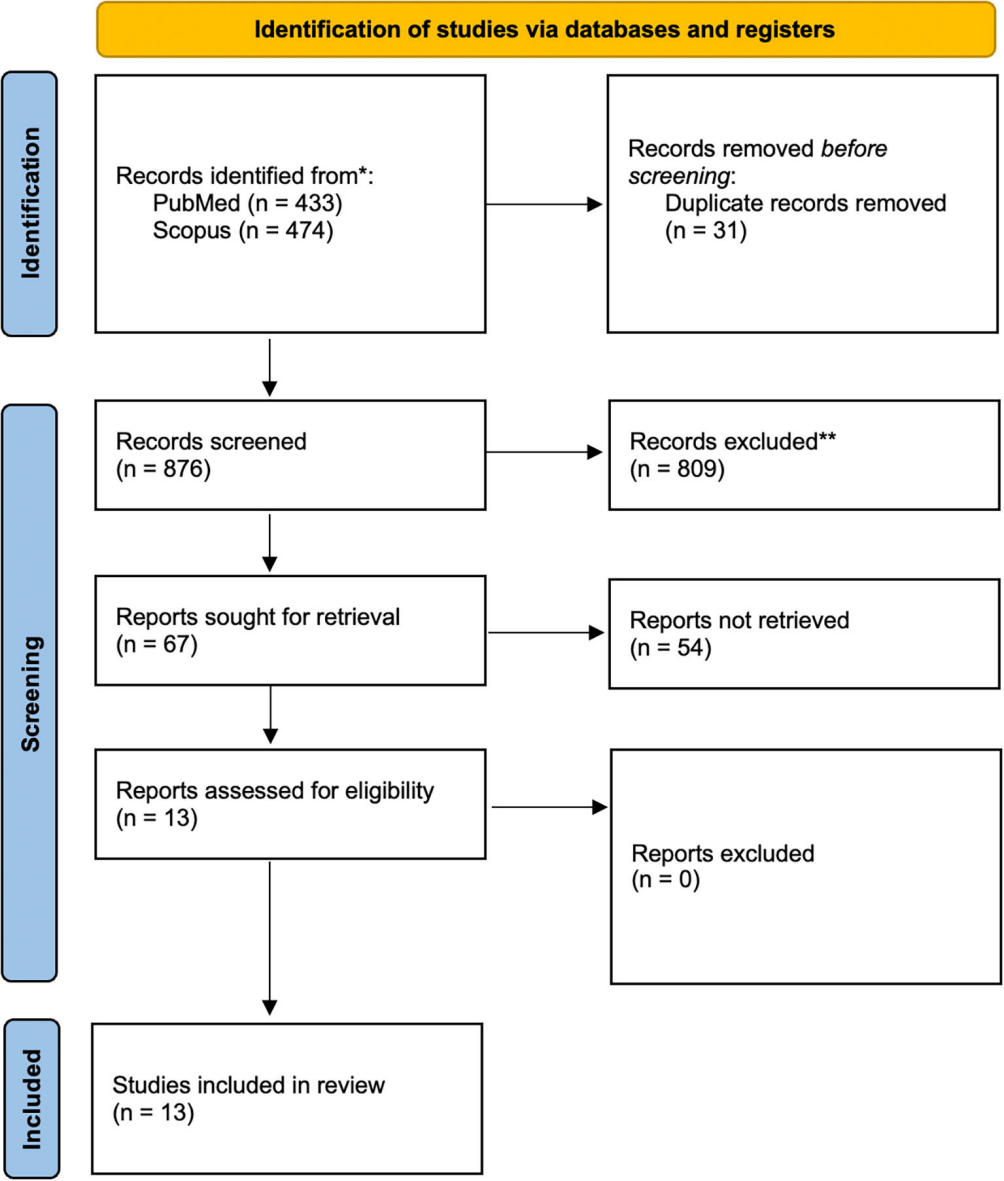
PRISMA 2020 flow diagram for new systematic reviews which included searches of databases and registers only.

As shown in Figure 1, a total of 907 records were initially retrieved from accredited databases, Scopus and PubMed. After removing 31 duplicate records, 876 studies remained for screening. Applying the PEO framework, 809 studies were excluded. Finally, 13 articles were ultimately included in the final review. These 13 studies ^14– 26^ comprised a total of 19,312 participants. The geographic distribution of the studies included three from Iran, two from China, two from the United States, and one each from France, Ethiopia, Sweden, Thailand, Brazil, and Japan. Additional characteristics of the participants and study designs are detailed in Table 2.

**Table 2.** Characteristics of included studies.

Author	Country	Design	Sample	Findings
Abdollahi, Agajani-Delavar, et al., (2016) ^31^	Iran	Cohort	838 postpartum women	Perceived social isolation, lack of marital satisfaction, and low self-efficacy as a parent are risk factors for postpartum complications
Abdollahi, Zarghami, et al., (2016) ^30^	Iran	Cohort	2.279 postpartum women	Psychological disorders during pregnancy, anxiety disorder, adequate parenting skills, higher marital satisfaction, frequency of more frequent rituals, and husband's education level are risk factors for postpartum depression
Bränn et al., (2017) ^28^	Swedia	Case-Control	291 postpartum women (63 cases and 228 control)	History of depression, anxiety disorder, education level is a risk factor for postpartum depression
Faisal-Cury (2021) ^23^	Brazil	Cohort	358 postpartum women	Low maternal confidence at 6-8 months after delivery and the type of vaginal delivery, education level and maternal confidence in later periods are risk factors for postpartum complications
Farhat et al., (2015) ^32^	Iran	Cohort	682 postpartum women	Multiple pregnancies, and advanced age are risk factors for postpartum depression
Hacker et al., (2022) ^22^	USA	Cohort	206 postpartum women	Gestational age at delivery, and early pregnancy BMI are risk factors for postpartum complications
Iwata et al., (2015) ^34^	Japan	Cohort	479 postpartum women	Emergency cesarean delivery, lower satisfaction with the childbirth experience, long-term complications with the newborn, more concerns about baby care after hospital discharge, and more concerns about personal life after hospital discharge are risk factors for postpartum depression
Klumpner et al., (2020) ^25^	USA	Cohort	7853 postpartum women	Gestational age, multiple pregnancies, diabetes mellitus and hypertension are risk factors for postpartum hemorrhage
Landman et al., (2024) ^15^	France	Cohort	3310 postpartum women	Childhood trauma, experience of stress during pregnancy, anxiety disorder, hypertension, education level, diabetes mellitus, multiple pregnancy, and family history of anxiety disorder are risk factors for postpartum depression
Malaju et al., (2022) ^20^	Ethiopia	Cohort	779 postpartum women	Anxiety disorders, lack of social support, low education level, working mothers, and fear of childbirth are risk factors for postpartum complications
Sittiparn & Siwadune (2017) ^29^	Thailand	Cohort	650 postpartum women	Age >35 years, pre-pregnancy BMI >25 kg/m ^2^, hypertension during pregnancy, DM type 2 are risk factors for postpartum hemorrhage
Wang et al., (2024) ^16^	China	Cohort	1077 postpartum women	Number of negative finger tests, postpartum hemorrhage, preeclampsia, diabetes mellitus, hypertension, education level, method of delivery, prenatal reproductive tract culture, and uterine exploration are risk factors for postpartum endometritis
Zhou et al., (2019) ^27^	China	Case-control	510 postpartum women (102 cases and 408 control)	Preeclampsia and postpartum hemorrhage are risk factors for postpartum venous thromboembolism

### Systematic review

Across the studies, a wide range of risk factors for postpartum complications were identified. Several studies consistently reported psychosocial and psychological factors such as childhood trauma, anxiety disorders, history of depression, lack of social support, low maternal confidence, low self-efficacy, perceived social isolation, and low marital satisfaction as significant contributors to postpartum depression and complications. ^14– 17, 22, 23^ Sociodemographic factors, particularly low education level and working status, were also frequently associated with increased risk. ^16, 22, 23^ Obstetric and clinical risk factors identified included gestational age at delivery, multiple pregnancy, preeclampsia, postpartum hemorrhage, emergency cesarean section, and method of delivery. ^19– 21, 25, 26^ Additionally, chronic medical conditions such as hypertension, diabetes mellitus, and high pre-pregnancy BMI were commonly reported across studies. ^22, 24, 25^ Cultural and contextual factors, such as parenting rituals and husband’s education level, were also noted as influential in some settings. ^15^ Overall, the findings demonstrate the multifactorial nature of postpartum complications, highlighting the interplay between psychological, sociodemographic, medical, and obstetric variables.

### Pilot Meta-analysis

**Anxiety disorders**

**Figure 2.**
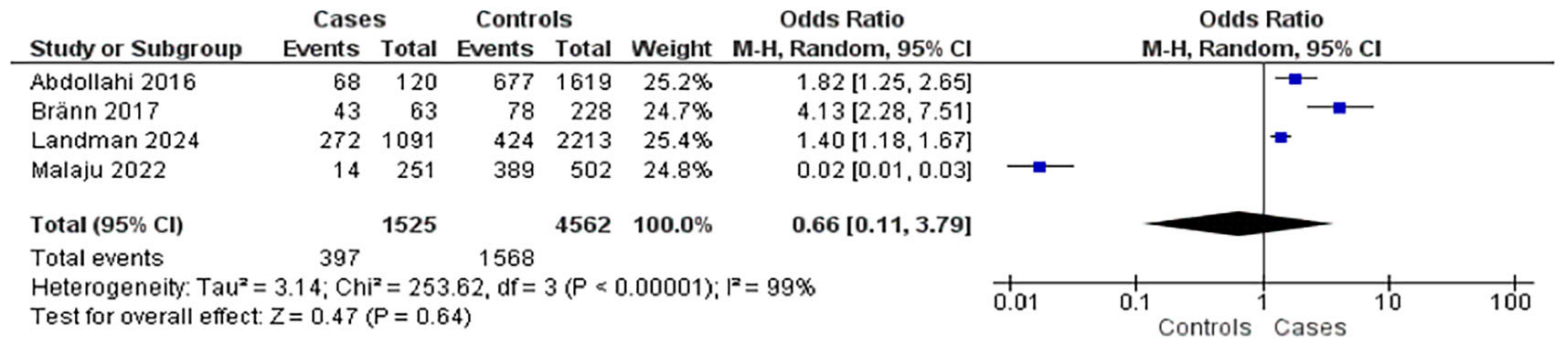
The influence of anxiety disorders on postpartum complications.

Four studies investigated the association between anxiety disorders and postpartum complications ( Figure 2). These included four case-control studies involving 1,525 mothers with postpartum complications and 4,562 controls. Overall, the presence of anxiety disorders was associated with a 0.66-fold increase in the risk of postpartum complications (OR = 0.66; 95% CI: 0.11–3.79; p = 0.64), although the association was not statistically significant. Substantial heterogeneity was observed across studies (I ^2^ = 99%).

**Education level**

**Figure 3.**
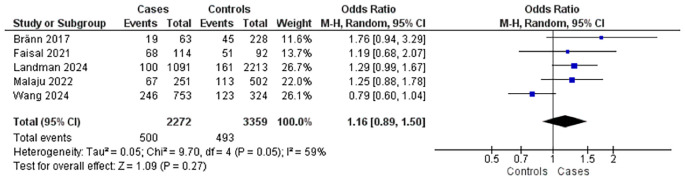
The influence of education level on postpartum complications.

Five studies explored the relationship between maternal education level and postpartum complications ( Figure 3). These comprised five case-control studies with 2,272 mothers experiencing complications and 3,359 controls. Findings indicated that mothers with lower education levels had a slightly increased risk of complications (OR = 1.16; 95% CI: 0.89–1.50; p = 0.27), the association was not statistically significant. Moderate heterogeneity was detected among the studies (I ^2^ = 59%).

**Hypertension**

**Figure 4.**
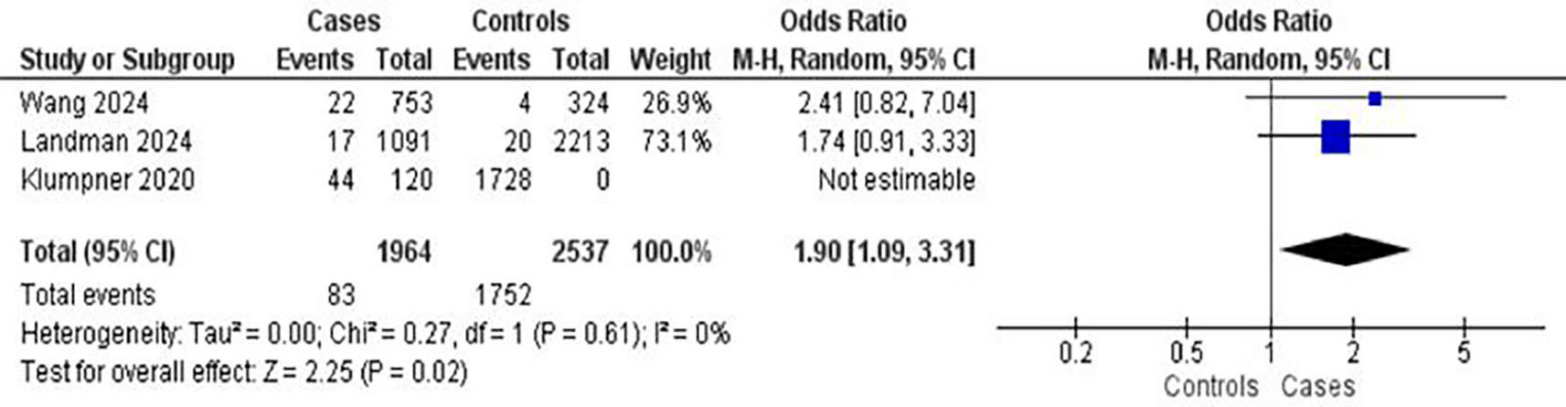
The influence of hypertension on postpartum complications.

Three studies examined hypertension as a risk factor for postpartum complications, encompassing 1,964 mothers with complications and 2,537 controls ( Figure 4). Hypertension was associated with an increased risk of complications (OR = 1.90; 95% CI: 1.09–3.31; p = 0.02). There was low heterogeneity among the studies (I ^2^ = 20%), suggesting relatively consistent findings.

**Diabetes mellitus**

**Figure 5.**
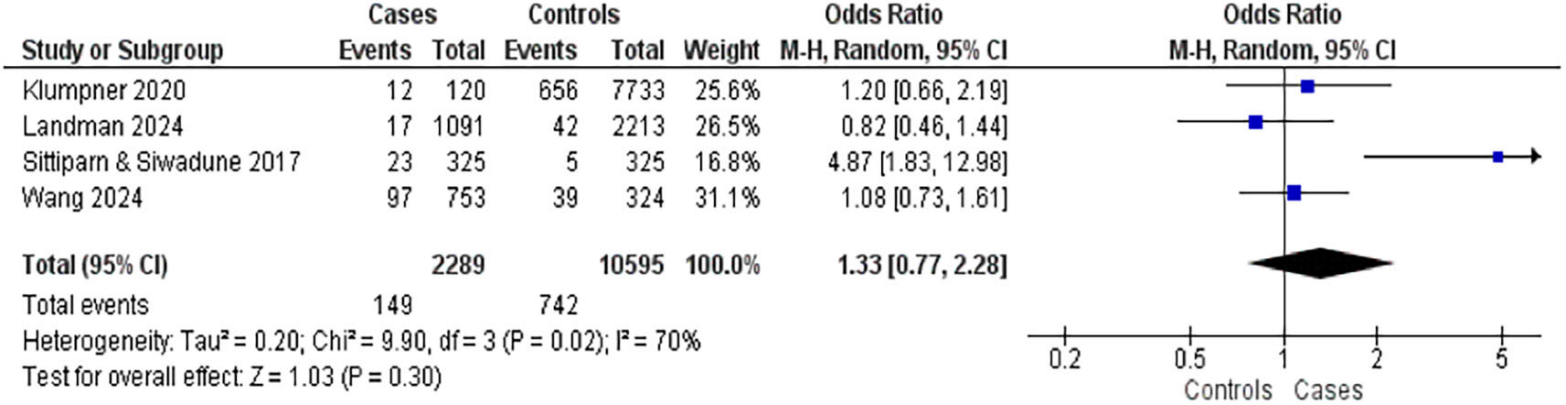
The influence of diabetes mellitus on postpartum complications.

Four studies reported on the relationship between diabetes mellitus and postpartum complications ( Figure 5). These included three case-control studies with 2,289 mothers who experienced complications and 10,595 controls. Diabetes mellitus was associated with a slightly elevated risk of postpartum complications (OR = 1.33; 95% CI: 0.77–2.28; p = 0.30), the association was not statistically significant. However, high heterogeneity was observed among the studies (I ^2^ = 70%), indicating variability in findings across the studies.

## Discussion

This review identifies multiple factors associated with postpartum complications, including postpartum hemorrhage, advanced maternal age, depressive and anxiety disorders, preeclampsia, multiple pregnancies, vaginal delivery, infant health problems, childhood trauma, low social support, low levels of maternal and paternal education, unplanned pregnancy, and marital conflict.

Postpartum hemorrhage is a major predictor of postpartum complications. One study in 2024 reported a p-value of 0.010 and a prevalence ratio (PR) of 2.63, indicating that mothers who experienced hemorrhage had a 2.63 times higher risk of complications compared to those who did not. ^21^ Hemorrhage contributes to a reduction in intravascular volume, leading to decreased venous return, cardiac output, and mean arterial pressure. The physiological compensation involves an increase in maternal heart rate through sympathetic activation and baroreceptor reflexes, which may further exacerbate postpartum complications. ^27^ Supporting evidence includes a report with a p-value of <0.05 and OR of 1.003, as well as another study showing a p-value of 0.003 and OR of 4.60. These findings emphasize the critical need for vigilant monitoring and immediate management of excessive bleeding after childbirth to prevent outcomes such as shock or organ failure.

Advanced maternal age has also been consistently associated with increased risk. Data from 2017 showed that mothers aged over 35 years were 2.703 times more likely to experience postpartum complications compared to younger mothers (p < 0.001). ^17^ Additional research in 2015 reported a similar trend with a p-value of 0.001 and an OR of 1.07. ^18^ This population is also at greater risk for postpartum depression, which affects 10–15% of women after childbirth. ^28^

Postpartum depression is itself a strong risk factor for complications. Meta-analytic evidence reports odds ratios of 2.26 (95% CI: 1.38–3.7) and 1.82 (95% CI: 1.25–2.64), respectively, highlighting a significant association between PPD and postpartum illness. ^30^ Further findings indicate an adjusted odds ratio (AOR) of 4.01 for individuals with a history of depression (p < 0.01), underlining the importance of recognizing prior mental health conditions. ^29^

Anxiety disorders also contribute notably to adverse outcomes. A study in 2023 ^30^ showed a p-value of <0.001 and an OR of 3.58, suggesting that anxiety increased the risk of postpartum complications by 3.58 times. ^28^ Some analyses conclude that anxiety is a more significant predictor of PPD than prior depression. Research in 2016 found that prenatal anxiety symptoms are more prevalent in women who later develop PPD. ^28^

Preeclampsia is another critical factor. A study in 2024 reported a p-value of <0.05 and an OR of 9.769, indicating nearly a tenfold increased risk of postpartum complications among mothers with preeclampsia. ^19^ Although typically observed during pregnancy, preeclampsia can persist or even manifest postpartum, warranting close monitoring of blood pressure and renal function in affected women.

Multiple pregnancies also increase postpartum risk. Data showed a p-value of 0.034 and an OR of 1.6, suggesting a 60% higher risk of complications in women with multiple gestations. ^22^ Another study in 2021 ^31^ found a stronger association (p = 0.024, OR = 3.6), emphasizing the elevated risk of postpartum depression by the 42nd day after delivery in mothers with twins. ^18^

Vaginal delivery, while often considered low risk, has been associated with postpartum complications. Evidence from 2024 revealed a p-value of <0.001 and an OR of 2.083, indicating a more than twofold increased risk compared to cesarean delivery. ^25^ Vaginal mucosal injury and the subsequent risk of infection are proposed as underlying mechanisms.

Infant health problems also contribute to maternal complications. A 2015 ^32^ study showed a significant association (p < 0.001), with low birth weight and neonatal morbidity being indirectly linked to maternal mental health issues, including PPD. ^33^ Close monitoring of the infant is essential to mitigate maternal emotional distress and ensure holistic recovery.

Childhood trauma has been identified as a long-term predictor of postpartum complications. Research in 2024 demonstrated a p-value of <0.001 and an OR of 1.3, linking past trauma—especially sexual abuse and neglect—with postpartum blues and depressive symptoms. ^22^

Social support plays a significant protective role. A 2022 study found that low social support was associated with an OR of 11.34 for developing PPD (p < 0.001), suggesting that women lacking emotional or instrumental support were over eleven times more likely to experience postpartum complications. ^14^ This underscores the value of postnatal support, especially from partners and family.

Educational attainment, both of the mother and the husband also emerged as key determinants. ^34^ One study found that low husband education was associated with an OR of 5.2 for postpartum complications (p < 0.001). ^35^ Another report identified low maternal education as a major risk factor, with an OR of 13.62 (p = 0.013). ^23^ Education level is often a proxy for socioeconomic status and access to health information, which may explain the observed associations.

Unplanned pregnancies contribute to increased risk as well. Research in 2018 ^36^ indicated a highly significant association (p = 0.00), and a 2023 study reported an OR of 1.36, suggesting increased complications in mothers with unplanned pregnancies. ^14^ Lower satisfaction with the childbirth experience and increased stress, especially among first-time mothers, may underlie this finding.

Marital conflict is a prominent psychosocial risk factor. A study in 2022 ^37^ reported a p-value of 0.004 and OR of 13.3, demonstrating a strong link between relational dissatisfaction and postpartum complications. ^14^ Poor partner support and unresolved conflicts can increase maternal vulnerability, particularly during the emotionally demanding postpartum period.

Given these findings, early screening and education about postpartum mental health are imperative. Healthcare professionals should be aware of the potential progression from postpartum blues to PPD and proactively offer guidance and support. Raising awareness of PPD symptoms and encouraging timely help-seeking behaviors may substantially improve maternal and infant outcomes.

Technological innovations also show promise in postpartum care. A 2022 study evaluated a digital blood pressure monitoring system using Bluetooth-enabled devices and mobile applications, which proved effective in early detection and intervention for postpartum hypertension. Such digital health interventions represent a valuable strategy for enhancing maternal health surveillance and timely care delivery.

Despite the strengths of this review, limitations should be noted. The inclusion period was restricted to studies published between 2014 and 2024, which may introduce publication bias. Nonetheless, the incorporation of data from both high- and low-resource settings strengthens the generalizability of the findings.

## Conclusions

Postpartum complications are influenced by a range of psychological, obstetric, and sociodemographic factors, as identified in this review. Key risk factors include postpartum hemorrhage, maternal age over 35, depressive and anxiety disorders, preeclampsia, multiple pregnancy, type of vaginal delivery, infant health concerns, childhood trauma, low social support, low educational attainment of both mothers and husbands, marital dissatisfaction, and perceived social isolation. While unplanned pregnancy and direct effects of marital conflict were not explicitly reported, related psychosocial stressors were evident. Although none of the included studies directly evaluated specific interventions, the findings underscore the importance of early detection and targeted management strategies. The implementation of risk assessment tools, routine screening for postpartum depression, and the integration of digital monitoring programs—particularly for conditions such as postpartum hypertension—may enhance early identification of at-risk women. Maternal and child health professionals, particularly midwives, should prioritize comprehensive monitoring from pregnancy through the postpartum period, with particular attention to psychosocial and medical risk profiles, especially in resource-limited settings.

## Data Availability

No data are associated with this article. Zenodo: Prisma Checklist, https://doi.org/10.5281/zenodo.15678965 ^38^ This project contains the following extended data: PRISMA_2020_checklist.docx Data are available under the terms of the Creative Commons Attribution 4.0 International license (CC-BY 4.0).
