# Supplementary material for: Factors associated with postpartum complications: A systematic review to inform early detection strategies
Source: F1000Res. 2025 Nov 22;14:649. Originally published 2025 Jul 3. [Version 2] doi: 10.12688/f1000research.166397.2 (PMC12612618; doi:10.12688/f1000research.166397.2)
Supplement: Peer review history

# Reviewer report: Reviewer response for version 1

**DOI:** 10.5256/f1000research.183378.r427938

**Published:** 2025-11-12

**Authors:** Fitriani Nur Damayanti

**Affiliations:** 1 Universitas Muhammadiyah Semarang, Semarang, Central Java, Indonesia

Artikel ini memiliki kualitas tinjauan sistematis yang baik, data berasal dari berbagai negara, sampel yang tersedia sangat memadai, dan orientasi implementasi (deteksi dini). Artikel ini sangat relevan dengan praktik klinis kebidanan, terutama dalam menyediakan skrining pascapersalinan, tidak hanya fisik tetapi juga mental dan relasional, serta penerapan pemantauan pascapersalinan digital (pemantauan tekanan darah jarak jauh telah terbukti efektif). Namun, masih perlu memperluas cakupan basis data dan menghubungkan hasilnya dengan rekomendasi intervensi berbasis bukti untuk deteksi dini.

Are the rationale for, and objectives of, the Systematic Review clearly stated?

Yes

Is the statistical analysis and its interpretation appropriate?

Yes

If this is a Living Systematic Review, is the ‘living’ method appropriate and is the search schedule clearly defined and justified? (‘Living Systematic Review’ or a variation of this term should be included in the title.)

Yes

Are sufficient details of the methods and analysis provided to allow replication by others?

Yes

Are the conclusions drawn adequately supported by the results presented in the review?

Yes

Reviewer Expertise:

Midwifery

I confirm that I have read this submission and believe that I have an appropriate level of expertise to confirm that it is of an acceptable scientific standard.

# Reviewer report: Reviewer response for version 1

**DOI:** 10.5256/f1000research.183378.r397879

**Published:** 2025-11-03

**Authors:** Eny Retna Ambarwati

**Affiliations:** 1 Sekolah Tinggi Ilmu Kesehatan Akbidyo, Yogyakarta, Indonesia

The research titled "Factors Associated with Postpartum Complications" is aligned with its objectives through a systematic review method. The study successfully gathered and analyzed 13 relevant studies. The findings clearly outline various clinical and psychosocial risk factors. The discussion elaborates on these findings, and the conclusion consistently summarizes that identifying these factors is crucial for early detection strategies. However, there are aspects that need to be re-evaluated concerning the consistency of terminology, such as "postpartum women" versus "postpartum period" or "postpartum disease complications" versus "postpartum complications.

Are the rationale for, and objectives of, the Systematic Review clearly stated?

Yes

Is the statistical analysis and its interpretation appropriate?

Yes

If this is a Living Systematic Review, is the ‘living’ method appropriate and is the search schedule clearly defined and justified? (‘Living Systematic Review’ or a variation of this term should be included in the title.)

Yes

Are sufficient details of the methods and analysis provided to allow replication by others?

Yes

Are the conclusions drawn adequately supported by the results presented in the review?

Yes

Reviewer Expertise:

NA

I confirm that I have read this submission and believe that I have an appropriate level of expertise to confirm that it is of an acceptable scientific standard.
